# Recueil de Memoires de Medecine, de Chirurgie, et de Pharmacie Militaires

**Published:** 1847-01-01

**Authors:** 


### Recueil de Memoires de Medecine, de Chirurgie, et de Pharmacie Militaires. Vol. LX. 8vo. pp. 390. Paris, 1846.

There are two or three papers in the new volume of these Memoirs of Military Medicine an account of which we think will prove acceptable to to our readers. The first of these is

### On the Climate and Diseases of Algeria. By Dr. Casimir Broussais.

Few lots are more deplorable than that of the soldier in French Africa. Forced by the tyranny of the conscription from his own fine country into one abounding in fever and other fatal disease, he finds himself engaged in a description of warfare of the most harassing kind, in which success is accompanied with neither honour, emolument, or a temporary cessation of toil and suffering. We read that, when the French soldiers were aware that Ambrose Paré, and in later times the excellent Larrey, was on the field of battle they entered into the engagement with zeal and eagerness, well knowing that, in the event of casualty, ample provision for it was at hand. What must be the reflections of the poor fellows in Algeria, knowing as they do too well that their hospital accommodation is wretchedly defective in quantity and quality, and that hundreds and thousands of their sick comrades perish in consequence of this and of the hasty removals the nature of the warfare carried on submits them to. Dr. Casimir Broussais, it is true, in this paper says little of these things. High in office, and remaining for a year only in charge of one of the hospitals at Algiers, where possibly all that was required was found, he probably had neither the opportunity of observing the full extent of these disasters, or the desire of speaking truths of this kind, which are found to be so unpalatable to the authorities at home. Communications, however, received in France from medical officers, actively engaged in the perils of the campaign, dilate upon the dreadful hardships they and the men under their care are subjected to, and the callous indifference and neglect with which their representations are received. Never was waste of life more wanton and unjustifiable than in this insensate and futile attempt at the conquest of Algeria!

In the mean time, however, we must not neglect any improvements in medical knowledge derivable from the consideration of these calamities: and we are paying the medical service of Algeria but a just compliment, when we say that it seems unanimously actuated by a fervid desire of benefiting the poor objects of its care and extending the bounds of medical science. Dr. Broussais' paper is an interesting one, and we will briefly glance at the most important points it touches upon.

The French medical officers have justly paid much attention to the *meteorology* of Algeria. It seems the maximum of *temperature* is not at 2 P.M. as in Europe, but at 11 A.M. The mean temperature of the year at 6 A.M. is 65° F. and reaching 72° by 10, it scarcely afterwards exceeds this. It gradually diminishes, and at 10 P.M. is nearly the same as at 6 A.M. The nights are not cold, the thermometer not sinking below 62° or 63°. How are we to explain then those remarkable transitions from heat to cold which are described as occurring during the passage from day to night in hot countries? These contrasts do not exist in the temperature when observed in the shade, but in the great differences of the temperatures taken in the sun and in the shade or at night. "Upon making the experiment I found the difference observed at the same hour to be enormous. Thus, when the temp. was 68° in the shade towards the north, and from 77° to 87° in the sun with free access of air, it was from 122°

to 142° in the sun, sheltered from the wind. It is to such transitions the soldier is exposed when on his march through the inequalities of a mountainous country." The *barometer* is generally high and liable to few oscillations. There are usually 200 fine clear days in the year, without cloud or rain; and but 50 wet or partially wet ones. When rain does come, however, it descends in true torrents. The wind blows ordinarily from the W. or NW. Storms are rare. The *summer heats* are of excessively long duration, four months passing without one drop of rain. The mean temperature in the shade is then about 82°, frequently rising to 85° and 95°, and but seldom to 105° and 113°. Without the sea-breeze this heat is insurmountable. It is under a privation of this, and towards the end of the summer, in September, or even October, that the terrible *Sirocco* prevails.

"As soon as it arises the heavens become of a grey or reddish colour, and the horizon is darkened. The air is loaded with dust, which is hurled along from a distance to the sea, above which it forms a greyish cloud. If a window is opened, air as hot as that from a furnace fills the room, imparting a pricking sensation to the body, and a burning one to the eyes. When you go out it is just as if you entered a heated stove. In vain do you open your mouth and make repeated efforts at inspiration. The air which enters your lungs seems only to suffocate you. You seek for the vital element, but it is not there. You exhaust yourself with vain efforts; your head is confused; you are as if intoxicated and overcome by an indefinable feeling of fulness, you are rendered unfit for every occupation or thought, insensible to every feeling but that of your own misery, I can only compare the indifference to oneself and others which is then felt, to that moral self-abandonment which sea-sickness induces.

"When this terrible wind arises suddenly and surprises a column on the march, the soldiers instantly stop, sink down as if under an intolerable burden, and remain on the route, notwithstanding the risk of being surprised by the enemy. More than once, one of these unfortunate fellows has become the victim of so intolerable a sense of uneasiness that he has become instantly disgusted with life, and has blown out his brains, before even his absence from the ranks was observed or his intention suspected.

"Sometimes the tempest of the sirocco becomes furious. It is then preceeded by a hollow rumbling, and rushes on with a frightful rapidity, roaring louder and louder, raising the sand like a water-spout, overthrowing every thing in its way, carrying away with it the roofs of houses, tents, and huts, uprooting trees, and leaving nothing standing. The animals fly for shelter, the camels bend their knees on the sand, and man casts himself down on the ground: for all that will not bend are destroyed. More than once our troops, marching in the large vallies open to the south, have encountered these disasters."

Whole clouds of locusts sometimes accompany the sirocco, surrounding and covering everything and person, utterly destroying the vegetation of the portions of country they pass over, until carried off by the violence of the winds which brought them. Ordinarily the sirocco soon passes away and those subjected to it quickly regain their usual state of health. It rarely continues an entire day; but during its prevalence pernicious fevers are easily generated, and the condition of the sick becomes dangerously aggravated—as also does that of those who seemed to have entered upon a prosperous convalescence.

The *Winter* in Algeria is to be dreaded rather on account of its humidity than its temperature. Snow is rarely seen, except in some of the mountains, and the rain only occurs at intervals, but then in immense quantities.

The abstemious habits of the Arab are well suited to the climate he inhabits. When the European arrives during the temperate season, abundant exercise and a delightful air excite a vigorous appetite. This he indulges not only with fruits and vegetables like the natives, but also with large quantities of animal food. Digestion being vigorous, all this seems easily disposed of; but when the hot weather arrives, nature warns us by the diminution of appetite to be more cautious, both as regards quantity and quality of food. She is too often unheeded, and the flagging stomach is excited beyond its powers by means of various stimuli, especially absinthium. Diarrhœas and dysenteries are produced, and if the regimen is not altered, become serious and persistent, and pave the way, by shattering the constitution, to the access of intermittent fever, which does not attack more than 1 in 30 of those who have not already suffered from flux. Owing, however, to greater care than heretofore, in this and other hygienic particulars, the mortality of the army has become reduced from 143 in the 1000 in 1840, to 60 per 1000 in 1843. The mere action of prolonged heat, however, suffices to enfeeble the constitution. The functions of every organ, except two, are diminished in activity, and these are the skin and the liver, the amount of the secretions from which causes a great loss to the economy, and implies an activity of the secretory organs which explains the occurrence of much of the disease of the skin, liver, and gastro-intestinal membrane. The great losses by secretion might be expected to give rise to an impoverished condition of the blood ; and, in fact, the physical appearance of the patient, the difficulty with which hæmorrhages are borne, and the chemical analyses of MM. Leonard and Foley, prove this to be the case.

*Diseases of Algeria.*—" If the pathology of Algeria has many points of contrast with that of France, it has likewise its own distinct phenomena. Thus, of a given number of men, there are a greater number fall ill in Algeria, and a larger proportion of these die, though much fewer now than heretofore; but what is most characteristic, is the striking correspondence of the diseases with the seasons, and the nature of each morbid manifestation. This is seen in extreme climates, and in Algeria, which is nevertheless intermediate between these and temperate ones. I was told that, as I went to Algiers in the winter season, I should only find there the same diseases as in France. This is a mistake. I visited the three military hospitals there in December, and was at once struck with the general aspect of the patients, presenting as they did different affections from those I had just left at Paris. I found a great number of miserable, exhausted, languishing frames, crowds of poor creatures undermined by diarrhœa or chronic dysentery, or swollen by dropsies. The countenance of such as were less seriously affected had that wan, yellow, or leaden appearance characteristic of those who have been the frequent subjects of intermittent fever. It was impossible for me not to recognise here the marks of the diseases peculiar to Africa, and it is not requisite to await the epidemic season to distinguish their prominent characters. By the side of these characteristic endemic types, were to be observed cases of sporadic diseases entirely analogous to those which I had treated a fortnight before at Val de Grâce."

At the commencement of the year the hospitals are filled with the cases remaining over from the hot season, and get gradually cleared as these die off or are discharged convalescent. It is not until June that the number of new patients notably increases, which it continues to do until August, when it attains its maximum. Official statements inform us, that the mean number of admissions into the entire Hospitals of Algeria for the first three months 1840-3 amounted to 13,720, and for the month of August alone to 11,809. The admissions in December were only 5,678. The same statements shew, also, that the number of sick exceed those of the effective force by one-fifth, so that 50,000 men furnish 60,000 patients per annum !—not that all the effective force enters the hospital, but that a certain number does so several times. The general mortality of the troops (1845) is 80 per 1000 on the sick, and 94 on the effective force—it having been 143 in 1840 and 60 in 1843.

The Diseases of Algeria have been properly divided into those which are *sporadic* and those which are *endemo-epidemic*; the former being the same as observed in Europe, the latter proper to the country. These last consist of intermittent and remittent fevers, diarrhœas and dysentery with hepatitis. Their prevalence commences in June and rapidly increases until September, when it attains its maximum. In his enquiries concerning the proportion of soldiers who resisted the endemo-epidemic influences, Dr. Broussais found that of 100 men entering the hospital, 20 had had fevers, 20 diarrhœa or dysentery, and 54 fever and either diarrhœa or dysentery, only 6 escaping all climacteric influence, and indeed only 3, for the other three had not yet passed a year in Africa. It is, indeed, scarcely possible for any one to live even a few months in Algeria without suffering more or less from diarrhœa and dysentery—so universal are their prevalence. Several, however, escape fever, even after more than a year's residence. Other persons, during the hot season, have attack after attack with little interval, and may carry the seeds of a relapse back with them to France. After repeated attacks, dropsy and other diseases result, which usually terminate in death ; but the recovery of some of these cases, on a return to Europe being allowed, has seemed almost miraculous.

*Dysentery and Diarrhœa.*—There were 367 cases of acute abdominal flux admitted into the Hospital in 1845, of which 162 were examples of dysentery and 205 of diarrhœa. The whole of the latter recovered, but 11 of the former died, being 66 per 1000, a vastly different proportion to the deaths from chronic dysentery, which amounted to 50 per cent. Among the cases the usual varieties of the disease were observed. "We are quite willing to designate these by the usual terms and call them inflammatory, bilious, pseudo-membranous, hæmorrhagic, and choleric or adynamic dysentery; but we see in them all but different forms and different degrees of one identical disease ; for in all of them we find the symptoms indicative of an internal phlegmasia, and in the fatal cases the marks of this colitis, while the treatment, with certain modifications, is the same in all." The treatment pursued was of the antiphlogistic character. A general bleeding was found of great service in severe cases, although its repetition was seldom required or would have been borne. Repeated local bleeding in the painful regions was very advantageous. To these measures were added hip-baths once or twice daily, small cold lavements of infusion of linseed and poppy-heads, or of simple water when the abdominal heat and thirst were great, tisanes formed of ricewater and gum, or lemonade and gum, and mild opiates; and the cure of the case was usually rapid. " When the disease was a *mild dysentery*, or mere *diarrhœa*, we contented ourselves with the regulation of regimen, and the supplementary treatment just detailed, without depletion. In these cases, the *sulphate of soda*, in doses of from 4 to 8 drachms *per diem*, was often advantageously given. This salt is indicated when a marked abdominal gurgling is heard, unaccompanied by heat or remittence, and in such cases it accelerates the cure. But, just like narcotics, it is powerless against a violent diarrhœa or dysentery, which, if given during the acute stage, it will only aggravate. In the treatment of that serious and so often fatal affection, *chronic dysentery*, a feculent regimen, emollients, and narcotics were resorted to. Flannel clothing was found of great service, as also were frictions of tartar-emetic ointment perseveringly applied. In spite of all measures, great numbers of the patients sank. In the analysis of the *blood* in *dysentery* by MM. Leonard and Foley, it was found in some cases to have retained its normal characters, but in most to exhibit an augmentation of fibrine, and a diminution of globules and of the solid materials of the serum. We regard these changes, with Andral, Gavarret, Becquerel, and Rodier, as consecutive; for they were never found at the onset of the disease, but only when it had made some considerable progress. As these changes resemble those produced in inflammation, they indicate that the dysentery should be treated as an inflammatory disease."

*Hepatitis and Abscess of the Liver.*—MM. Hassel and Catteloup have shewn the intimate connexion of hepatitis and abscess of the liver with dysentery. (*See Med.-Chir. Rev.* Jan. 1846, p. 65.)

"We firmly believe in the reality of such coincidence, which has in fact nothing surprising in it, since we have seen, on the one hand, the climate influences undue increase of the biliary secretions, and, on the other, the intestinal canal is disposed to diarrhœa. We, have endeavoured to shew, in a memoir upon this subject published in the *Journal de Medecine, Aug.* 1845, that by percussion in the hepatic region, and consideration of the seat of pain and of the disordered state of the biliary functions, we may often arrive at a diagnosis of hepatitis, which a superficial examination would have overlooked. But we must admit that, with every care, the most attentive practitioner may sometimes be surprised at the discovery after death of a hepatic abscess which was not suspected during life. Among the 686 fever patients admitted during 1845, there were 43 cases of hepatitis, 5 of whom had abscess. The deaths amounted to 7; but this high mortality would give a false idea of that of hepatitis in general, if we did not bear in mind that many patients who were admitted for fever, diarrhœa, or dysentery, were affected with various degrees of hepatitis which terminated by resolution."

*Intermittent and Remittent, and Pernicious Fevers,*—Dr. Broussais' observations under this head extend to a considerable length; but, as we treated somewhat at large upon the fevers of hot climates in our last number, we will confine our attention to one or two points in the present one. The author arrives at these conclusions. " 1. That intermittent and remittent fever prevails endemically in Algeria, and in Summer assumes the epidemic form. 2. That it is infinitely more frequent, more severe, and more fatal in marshy localities. 3. That the cause, whatever it may be, which induces it, exerts its action first upon the nervous centres, and from thence upon the other organic apparatuses, especially upon that of digestion."

In slight cases of intermittent fever, Dr. Broussais has found, both at Paris and Algiers, the administration of *cold lavements*, either with or without the use of quinine, an excellent means. They seem even to have the effect of preventing an access of fever which the heat of skin, especially of the abdomen, headache, uneasiness and loss of appetite, proved to have been imminent. For such patients it has often sufficed to diminish the amount of their food and administer a few cold injections, to entirely restore their health in a few days.

In respect of the treatment of the worst forms of remittent or pernicious fever, we have the following remarks :—

"According to what I have seen and heard in Algeria, most of the French practitioners have recourse to general and local bleeding; some, however, employing it with confidence, and relying much upon its association with an anti-periodic medication, and others resorting to it with reluctance, and with much doubt as to its utility. It soon became evident to me, upon my arrival in Africa, that not only is bleeding useful in intermittent and especially remittent fever, but that there is no disease or country in which it is of such efficacy as in these Algerian fevers. It, so to say, relieves the disease in a few hours, save where there is serious inflammation or changes of structure, and it forms an admirable preparative for quinine. I have frequently seen this drug quite inoperative until some leeches or a bleeding have removed the complication which proved an obstacle to success. Notwithstanding the great efficacy of bleeding, I have not resorted to it in all cases; but, when the patient has presented a weak faltering pulse, and other signs of collapse, I have at once ordered him a large dose of quinine, often with æther, employing revulsives at the same time. These last are highly useful in the comatose and algid forms of the disease, but only as adjuncts to other measures. As to *quinine*, it has operated wonders, whether given alone or in combination with bleeding. When an inflammatory complication has existed I have administered it immediately after the depletion, without waiting for the moistness and natural colour of the tongue being restored. The loss of blood seemed to enable the patient to pass at once from a state of disease to one of comparative convalescence; and the quinine prevented or moderated the violence of the symptoms, which always manifested their tendency to return. Upon these points I may refer to the practice of Johnson and Twining in India, and the success which followed their free use of the lancet."

*Sporadic Affections.*—The only one of these Dr. C. Broussais dwells upon is *phthisis pulmonalis.* In 930 patients admitted from the 1st January to the 1st November, only eight cases of pulmonary tubercles presented themselves, giving one for 116 patients; a proportion small indeed compared with that observed in the military hospitals of France, where a case of consumption shewed itself in every 41 patients, during 1831-44. Again, in 41 deaths, 2 only from phthisis are met with, or 1 in 20; while the proportion in Paris is 1 in 5.

"This disease is then, without any doubt, much less frequent in our African possessions than in France, and so great is the difference that it evidently depends on the climate, no secondary circumstance being capable of producing it. But, to render the difference still more striking, we must take into account the autopsies at which, the patients having sank under different diseases foreign to those of the chest, tubercles are nevertheless met with. Common as is this case at Paris, we only met with three examples in Algeria. Still, rare as is phthisis, it does occur in Algeria, and may originate there. And not only may it do so, but also, in spite of a prolonged residence, the vicinity of marshy emanations or the occurrence of numerous attacks of fever. The *antagonism* announced by Dr. Boudin is, in our eyes, an ingenious hypothesis, but, being destitute of foundation, it crumbles before exact observation. To complete this subject, we have to state the influence of the climate of Algeria upon the progress and cure of phthisis. Is its progress more rapid in Algeria? Certainly not, according to our documents. Is there a greater chance of cure than in France? The same documents reply affirmatively; for we have observed cures of circumscribed tubercle, and extraordinary amelioration in suppurating tubercle."

### Researches on the Condition of the Blood in the Endemic Diseases of Algeria. By MM. *Leonard* and *Foley*.

This forms an interesting complement to the paper just considered, and consists in a detailed account of numerous analyses of the blood in the *febrile diseases of Africa,* conducted in the same manner as those of MM. Andral and Gavarret were performed. Comprehensive tables, exhibiting the amount of the various component parts of the fluid, are given; but we can only find space for the general recapitulation of results.

"1. The *fibrine* is found in its natural proportions at the commencement of the fever. 2. It is diminished by the influence of duration and relapse of fever. 3. The passage from intermittence to remittence, from the continuous to the pernicious form, does not produce an increase or diminution of the quantity in any constant manner. 4. The amount is somewhat increased if there be complications, leading to the conclusion that these are of an inflammatory character. 5. The congestion of organs which takes place under the influence of fever may in some few cases proceed to the extent of inflammation, and in this way give rise to an increase of fibrine. 6. The engorgement of the spleen, which is observed in intermittent fever, is only exceptionably coincident with a defibrinated state of the blood, contrary to what is met with in the typhoid state. 7. The proportion of *globules* is only exceptionally increased, its tendency being to remain stationary or diminish. 8. The diminution only takes place under the influence of the prolongations of the disease, relapses, and an enfeeblement of the constitution. 9. Although the instances of increase usually occur in the severe forms of the disease, we cannot establish a relationship between these two circumstances. 10. The *solid matters* of the *serum* have a tendency to diminish in quantity, and this equally with the organic and inorganic materials. 11. The diminution of the proportion of *albumen* is very marked; but does not take place to the profit of the fibrine or the globules. 12. The proportion of *water* is diminished only in some very rare cases. It generally is increased, and often very markedly so. It is so almost always at the expense of the globules.

"To what reflections do our researches give rise? It cannot be denied that the blood is submitted to modifications in its *crasis* during the course of these fevers. These modifications are such, that, interpreted as by pathologists who have preceded us in the examination of other diseases, they will explain the morbid phenomena observed at a certain period of the pyrexiæ, (viz. those which succeed to the acute stage, preserving only the febrile periodicity, which indeed may also have ceased,) and are exhibited in chloro-anemia, anasarca, ascites, scorbutus, nasal hæmorrhage, &c. The impoverishment of the elements of the blood, considered separately as to the effects each exerts upon the organism, exhibits itself, as regards the *globules*, by the general debility, the decoloration of the skin and mucous membranes, and certain disorders of the circulation: the *fibrine*, by the violet spots of the skin, the epistaxis, the scorbutic bleeding from, and sometimes the gangrene of, the mouth, and the muscular pains of the limbs: the *albumen*, in puffiness, serous infiltration, ascites, and perhaps that serous diarrhœa which, in persons exhausted by fevers, almost always terminates the above-named disorders of the economy. * * * * * * * * * * * * * We must admit as conclusions—1. That the vitiation of the blood, such at least as it has been exhibited in our researches, can only be looked upon as consecutive, and as an effect of the disease. 2. That this vitiation, which is also manifested in other diseases as a consequence of their duration, as the beautiful researches of MM. Andral, Gavarret, Becquerel, and Rodier have taught us, presents no peculiarity in this description of affection, only inasmuch as it implicates, at the same time, a greater number of the element of the fluid. 3. That if the development of the fever is the result of the poisoning of the blood, which we do not absolutely deny it may be, the presence of the principle causing it is yet to find, and we may just as reasonably attribute, in the meantime, the earliest symptoms indicating the existence of the disease, to a morbid modification of some of the apparatus of the nervous system. 4. That to a certain degree the same reproaches may be addressed to the humoral pathology as to the anatomical pathology of the solids; that it fails to entirely raise the veil which conceals the essence of the disease from our view. 5. That notwithstanding this, the knowledge of the change or the proportion of several of the elements of the blood, operated by the influence of diseases, should be looked upon as a real addition to that of the lesions which the solids undergo; for it serves in some cases to clear up and explain the pathology of these latter. 6. That after the study of the fluids of the animal organism should come another, that of the laws by which that power which governs all the other systems of the economy acts. Little does it matter whether we call it vital power, innervation, plastic force or vital dynamism, provided it be examined by the procedures of philosophical experiment. The following sentence of Hippocrates will then receive its confirmation. 'We must consider in man not only the containing parts or solids, and the contained parts or liquids, but especially the active powers or the cause of vital movement.' "

### Cases of Wounds and Diseases of the Brain which tend to prove that the Faculty of Language is placed in the Anterior Lobes of that Organ. By M. *Bonnafont.*

In the *first* of these, a musket-ball entered the anterior portion of the left temple, and passed out at the same point on the opposite side, having traversed the anterior lobes of the brain. Some of the cerebral substance passed out at the apertures. The soldier, at first insensible, soon recovered his faculties, except that of smell, and the power of articulation. He preserved his memory of things and facts, as well as his hearing, as was manifested by the signs he made upon various questions being asked him. Five hours after he became comatose, and died 18 hours after the receipt of the wound. In the *second* case the ball traversed the right anterior lobe from below upwards, as well as the upper portion of the opposite lobe. The wounded man heard what was addressed to him, and at every question made a kind of groan, but he had entirely lost the power of speech. He died the next day. *Case* 3.—A man about 50 years of age was admitted into the Nantes hospital as insane, and, although speaking with difficulty, he could make himself understood, but his embarrassment of pronunciation increased, so that for 18 months before his death he was obliged, excepting two or three words, to express everything by signs. At the autopsy, an osseous tumour, the size of a large nut, was found in the left coronal fossa, which by its pressure had caused the disappearance of nearly the entire corresponding lobe of the brain, and a strong depression in the opposite one. The *fourth* case was that of a woman (æt. 55), who for several years had lost the power of speech, except for one word, which she incessantly repeated. At the autopsy, the two anterior lobes were found nearly atrophied and surrounded by a large quantity of fluid. The *fifth* case relates to the loss of memory properly so called, and not to the loss of the faculty of language. A sergeant was struck by a ball on the right parietal bone, its course seeming afterwards to be downwards and forwards towards the base of the brain. The attendants were surprised at hearing the wounded man reply at random to questions which were addressed to him. The reporter found he answered correctly enough when the question required no exercise of memory; but, in regard to anything which had happened, even a few days before, he made vain efforts to recollect himself. He continually exclaimed, "How strange it is that I should have lost my memory." He died the next day: but the urgency of affairs prevented an autopsy being made. *Case* 6.—A soldier, æt. 22, was struck on the right temporo-parietal region by some fragments of rock during the explosion of a mine, which drove the bone in upon the contents of the cranium. He remained some days unconscious; and on coming to himself he was deaf and partially blind, and had lost his memory to such an extent as scarcely to recollect the cause of his accident. The sight was gradually restored, as also, by the aid of galvanism, the hearing; but his memory, which was once very excellent, so as to enable him to commit long passages to it, and though now (eight months) somewhat improved, continues very defective, so that he is obliged to make great efforts to recall any event which but the evening before had made a great impression upon him. Oftentimes he cannot recollect for very short periods what has been said to him, however great the efforts to this end he may make. His speech is fluent, but certain words, which he pronounced well prior to his accident, are now articulated with difficulty. The consciousness of his state much annoys this patient, for he can write words correctly which he cannot pronounce without great difficulty.

### On Foreign Bodies in the Ear. By M. *Latour*.

These are numerous, and are naturally divided into two classes, those which are developed in, and those which are introduced into, the meatus. The latter class may be subdivided into bodies possessing spontaneous movements (insects): 2. bodies susceptible of swelling or even germinating in the ear (peas, &c.): 3, bodies of an invariable size (leaden balls, &c.): 4, liquid substances, capable of exerting a chemical action on the parietes of the meatus (acids, &c.) These divisions are not useless, and are much the same as those adopted by Paulus Ægineta and Ambrose Paré.

The *symptoms* are too obvious for transcription, in spite of which, however, the *diagnosis* is not always correctly made. A case is cited in which a fruit-stone has been mistaken for a sequestrum of the temporal bone; while at other times the canal has been needlessly irritated in searching for a foreign body which it did not contain. M. Velpeau relates a case in which the worst consequences resulted from this. Such occurrences will seem less strange when we recollect that few medical men habituate themselves to the exploration of the meatus. This would be unpardonable neglect in the military surgeon, seeing the frequency with which this part is made the seat of fraudulent attempts. The usual directions given for inspecting the ear are valueless, and nothing but the *speculum* will suffice in most cases. Its utility has been long recognized: for Fabricius Hildamus gives us a figure of one little differing from that now in use. M. Latour, however, considers the latter as too voluminous, and has contrived a simpler, smaller, and less expensive one, which can be put into an ordinary pocket-case. The sitting position is the best for its employment. The pavilion of the ear is to be drawn backwards, upwards, and outwards by the left hand, and the speculum gently introduced to the depth of from five to seven lines by the other, according to the length of the cartilaginous portion, the valves of the instrument rarely penetrating so far as the osseous portion. These are then expanded as much as possible and the direct rays of the sun, if obtainable, admitted. If not, we must have recourse to artificial light, employing reflecting lamps, or a silver spoon to concentrate the light. The author has frequently concentrated the rays of a candle or lamp by means of a lens. However obtained, the light should always be directed obliquely on the valve of the speculum, for the eye cannot otherwise explore the canal. If the wax is sufficiently abundant to intercept the rays of light it must be removed by the aid of sweet-oil and warm-water injections, or, if it is solid, by means of long thin forceps, having a piece of cotton on their extremities. When we find the canal obstructed by a foreign body, its nature may be often ascertained by *catheterism* of the meatus, which indeed is the only procedure admissible when the canal is anormally narrow, or its mucous membrane much swollen. In this last case, leeches, emollients, or prepared sponge, must be employed for the dilatation of the passage, according to circumstances.

*Prognosis.*—The presence of a foreign body in the ear is always a serious circumstance. The present disorder of hearing may become permanent. Besides the local symptoms which, and especially the pain, sometimes put on an alarming character, we have always to fear those dreadful effects of sympathy producing encephalic complications, of which so many examples are on record. These complications are of different kinds. Sometimes there is inflammation of the brain or its membranes; sometimes the accidents are of a nervous character, inducing epilepsy or convulsions; and at others, an unusual stimulus is imparted to the salivary glands and a profuse ptyalism brings on a state of marasmus. M. Lallemand believes the affection of the ear induces that of the brain, not only by propagating the diseased action by contiguity, but by maintaining a constant state of congestion near the cranium. In stating an opinion a number of circumstances must be taken into consideration, such as the irritability of the patient, his predisposition to cerebral affections, the nature of the foreign body, the degree of tumefaction, &c. &c. Thus, serious consequences have followed the movements of a flea in the meatus, and a roll of paper having penetrated the membrana-tympani, induced a fatal meningitis ; while, on the other hand, Larrey and others relate examples of large bodies, such as cherry-stones, teeth, &c, remaining in the meatus without causing the slightest pain.

*Treatment.*—Nowhere is the aphorism of Hippocrates, *sublata causâ tollitur effectus*, more applicable than here, provided it be enforced promptly. A great variety of means of extraction, according to the nature of the foreign body, have been resorted to. In all cases we should at once inject oil or some emollient fluid, which relieves the pain, facilitates the future extraction, and if the body be an insect it may cause its removal. M. Latour describes the various instruments which have been used in ancient and modern times. He speaks highly of the probe in the form of an ear-pick employed by M. Begin. When the body is forcibly fixed, he believes the use of a small wimble may be sometimes advantageous. A variety of forceps have been employed, and they answer excellently for pointed, angular, &c. bodies. Rounded bodies they seize with difficulty, and retain with as much, unless their extremities are concave. To be truly useful, each branch should be separately applied to the body and then locked like midwifery forceps.

Of foreign bodies which are developed within the ear itself, the *indurated cerumen* is the most common. The meatus in the young and in the adult is much moister than in the aged, in whom these concretions generally occur. These may vary much as respects consistence, colour, form and dimensions. If the concretion is soft it may be removed by an ear-pick ; but if indurated, the membrana-tympani might be injured by this procedure, and injections are to be preferred. M. Latour prefers oil to any other fluid, as partly dissolving the cerumen and facilitating the expulsion of the rest. Many cases of deafness, attributed to other causes, and long treated by other means, may, in this simple manner, be relieved.

*Insects* entering the ear at first cause a disagreeable sense of tickling, which is eventually converted into most dreadful pain, which may give rise to severe nervous symptoms, and even endanger the patient's existence. The earwig is considered by M. Latour, as well by other writers upon the subject, as the most dangerous of the insects which infest the ear. Many means have been recommended for the expulsion of insects. Various forms of probes and forceps have been contrived, the points of which are to be smeared with some sticky substance to secure the animal. Fluids for the purpose of impeding its respiration or poisoning it have been injected, such as sweet oil, milk, warm water, bitter almond oil, decoction of peach leaves, soap and water, essence of turpentine. Lastly, substances have been held at the external ear to tempt the insect out. "As to a little insect," says Ambrose Paré, "we may extract it by laying half a sweet apple at the orifice ; for the little creature, in attempting to nibble it, may be seized hold of." Laschevin says the half of a potatoe is a special antidote in this way for the ear-wig. M. Berard and other surgeons have obtained the exit of the larvæ of flies by employing a piece of meat ; and, according to M. Velpeau, milk attracts the earwig. All these means are desirable to be known, as in an emergency we may not have access to that one which we prefer: but, in the majority of cases, a simple injection of oil is all the treatment required.

We need not follow the author through the details he enters upon according to the nature of the obstructing body. He relates some interesting cases in illustration of the length of time foreign bodies may have remained in the ear prior to extraction. In two of these they excited more or less ptyalism, and in one lachrymation.

